# CrossInteraction: Multi-Modal Interaction and Alignment Strategy for 3D Perception

**DOI:** 10.3390/s25185775

**Published:** 2025-09-16

**Authors:** Weiyi Zhao, Xinxin Liu, Yu Ding

**Affiliations:** 1 College of Automation, Nanjing University of Information Science and Technology, Nanjing 210044, China; zhaoweiyi2023@126.com (W.Z.); dingyu@nuist.edu.cn (Y.D.); 2 Jiangsu Collaborative Innovation Center of Atmospheric Environment and Equipment Technology (CICAEET), Nanjing 210044, China

**Keywords:** LiDAR and camera fusion, LiDAR sensors, feature-level fusion, transformers, multi-modal perception

## Abstract

Cameras and LiDAR are the primary sensors utilized in contemporary 3D object perception, leading to the development of various multi-modal detection algorithms for images, point clouds, and their fusion. Given the demanding accuracy requirements in autonomous driving environments, traditional multi-modal fusion techniques often overlook critical information from individual modalities and struggle to effectively align transformed features. In this paper, we introduce an improved modal interaction strategy, called CrossInteraction. This method enhances the interaction between modalities by using the output of the first modal representation as the input for the second interaction enhancement, resulting in better overall interaction effects. To further address the challenge of feature alignment errors, we employ a graph convolutional network. Finally, the prediction process is completed through a cross-attention mechanism, ensuring more accurate detection out- comes.

## 1. Introduction

In recent years, the autonomous driving industry has developed rapidly. Object detection is becoming a vital process for the autonomous perception system. However, traditional 2D object detection methods, which rely solely on single-view images, fail to provide essential 3D information such as spatial distances. In contrast, 3D object detection techniques [1] offer a comprehensive understanding of the vehicle’s surroundings by assigning category labels and constructing 3D bounding boxes to capture object shape, distance, and direction. This capability is fundamental for tasks such as motion prediction and collision avoidance. To achieve reliable target detection, the simultaneous deployment of LiDAR and camera sensors is necessary. LiDAR provides essential localization and geometric information through low-resolution point clouds, while high-resolution RGB images from cameras offer detailed appearance information. This sensor fusion approach leverages the complementary strengths of both modalities, ensuring robust performance even if one sensor fails due to adverse weather conditions. Therefore, cross-modal information fusion is crucial for enhancing 3D object detection performance.

Existing multi-modal 3D object detection methods typically employ a modal fusion strategy that combines representations from different modalities into a hybrid form. For instance, early fusion methods such as PointPainting [2] integrate point cloud data by projecting them onto the output of an image semantic segmentation network. This process involves assigning category scores to each point based on the semantic information derived from the image segmentation, thereby enriching the point cloud with additional classification details. Similarly, MVP [3] (multi-modal virtual point) employs multiple 2D detection networks to create dense 3D virtual point clouds, enhancing the sparse original point cloud. While these virtual points can be integrated with traditional LiDAR points, the sparsity of the data can adversely affect fusion quality, leading to semantic information loss during projection from camera to LiDAR. Moreover, minor calibration errors between sensors can further compromise this approach. Recent deep fusion methods, such as Deepfusion [4] and BEVFusion [5] (Bird’s Eye View), have made strides by merging image and point cloud features into a joint BEV representation. Feature fusion has always been a focus of research in autonomous driving perception. Images and point clouds are presented in different views (i.e., camera view and real 3D view), and the semantic information they contain is heterogeneous, making it difficult to find point-by point-correspondence. This semantic alignment ambiguity is one of the biggest challenges in point cloud image multimodal fusion technology.

Most current multimodal fusion strategies ignore the unique feature information of each modality, ultimately hindering further improvement in model performance. Meanwhile, it is crucial to maintain modality-specific information and enhance other modality features during modal interaction. To overcome these limitations, we build a novel modality interaction strategy for multi-modal 3D object detection. In contrast to approaches like DeepInteraction [6], which utilize parallel representations for modal interaction, our core innovation involves an initial enhancement of modality-specific representations through a sequential interaction process. This is followed by a second interaction phase, where the augmented representation is utilized to derive another modality-specific augmented representation. This strategy effectively facilitates cross-modal interactions while capitalizing on the unique strengths of each modality.

Our contributions can be summarized as follows:

(1) A novel interaction framework is introduced that directly tackles the shortcomings of previous modal fusion techniques, thereby improving the robustness and accuracy of 3D object detection.

(2) Graph convolutional networks are employed to achieve effective feature alignment between the two modality-specific representations. This method enhances the interconnectivity of the features, ensuring that the complementary information from each modality is optimally integrated.

(3) A cross-attention mechanism is implemented to finalize the predictions process, ensuring that the synergistic interactions between modalities are effectively leveraged. This mechanism enhances the accuracy of 3D object detection tasks by enabling the model to focus on the most relevant information across modalities.

## 2. Related Work

### 2.1. Camera-Based 3D Perception

By consolidating information from multiple sources, BEV enables a more thorough understanding of the environment, thereby improving the accuracy and effectiveness of object detection in complex scenarios. There are two approaches: LSS-based explicit characterization [7] and Transformer-based implicit BEV characterization [8]. The Lift, Splat, Shoot (LSS) [7] approach extends the Orthographic feature transform (OFT) [9] by introducing latent depth distributions during the BEV feature pooling process. BEVDet [10] marks a pioneering effort in combining LSS with LiDAR detector heads, utilizing LSS to extract BEV features while employing LiDAR heads to predict 3D bounding boxes. Following this, BEVDet4D [11] enhances the model’s capabilities by incorporating temporal frames for velocity prediction. Meanwhile, BEVDepth [12] improves depth perception by using LiDAR to generate depth truth maps, whereas BEVStereo employs temporal stereo modeling to achieve more accurate depth estimations [13]. On the other hand, DETR3D [14] extends the DETR framework [15] for 3D applications, eliminating the need for explicit BEV feature generation and directly producing 3D bounding boxes through a Transformer-based architecture [8]. PETR [16] introduces a novel fusion technique, combining perspective feature maps with 3D position-embedded feature maps at an elemental level. Furthermore, BEVFormer [17] employs spatial cross-attention to facilitate view transformations and temporal self-attention for effective temporal feature fusion.

### 2.2. LiDAR-Based 3D Perception

Voxel-based methods effectively transform irregular point clouds into 2D or 3D compact grids, which are then compressed into BEV representations. This transformation simplifies the representation of point clouds and enhances overall detection performance. Notable examples, such as VoxelNet [18] and SECOND [19], leverage these BEV representations, which reduce scale ambiguity and minimize occlusion. Consequently, these make them particularly effective in complex environments. In parallel, point-based methods have advanced through the development of point set deep learning techniques, including PointNet [20] and other point cloud feature learning networks [21,22]. While voxel-based detectors are recognized for their high accuracy and relatively low latency, point-based methods excel in preserving geometric information from point clouds. However, this preservation often comes at the cost of increased computational complexity.

### 2.3. Multi-Modal Fusion-Based 3D Perception Methods

Fusion methods can be categorized into three key types based on the timing of the fusion process: early fusion, mid-level fusion, and post-fusion. Early fusion involves integrating data from different modalities during the data preprocessing phase. This approach aims to create a unified representation before any deep feature extraction occurs. A prominent example is PointPainting [2], which combines LiDAR data with image information from the outset. Similarly, PointAugmenting [23] recognizes the limitations of relying solely on semantic scores. It proposes that augmenting LiDAR points with deep features extracted from a 2D object detection network, influenced by camera images, can enhance the quality of the data representation. VBRFusion [24] proposes a two-branch structure that acts on the point cloud voxelization to aggregate high-dimensional features.

In contrast, mid-level fusion occurs at an intermediate stage, focusing primarily on the fusion of deep features after initial extraction. This method is often termed deep fusion and is currently regarded as one of the most promising strategies due to its ability to leverage deep learned representations. For instance, TransFusion [25] defines object queries in 3D space, integrating image features directly into these proposed bounding boxes, exemplifying proposal-level fusion. ContFuse [26] improves the fusion process by combining multi-scale convolutional features on a pixel-wise basis through successive convolutions. ConCs-Fusion [27] utilizes a method called context clustering-based radar and camera fusion for 3D object detection. Techniques utilized by DeepFusion [4], such as InverseAug and inverse rotation, further ensure precise alignment between LiDAR points and image pixels. BEVFusion [5] employs a local shared attention mechanism to project image features into the BEV space, effectively linking them with LiDAR features to produce a cohesive fused representation. BEV-CFKT [28], proposed in this paper, leverages knowledge transfer through Transformers for LiDAR-Camera fusion in the BEV space. RoboFusion [29] utilizes the SAM (Segment Anything Model) to develop the SAM-AD for autonomous driving scenarios, improving perception performance in complex environments through an adaptive fusion mechanism. GraphBEV [30] enhances the projected depth and adjacent depths from LiDAR to camera by building graph-based neighborhood information, addressing the local and global misalignment of inaccurate depth between LiDAR and camera BEV features. Fast-BEV [31] uses a Fast-Ray transform to efficiently project features to multi-scale BEV. PF3Det [32] combines a basic model with prompt engineering, enabling the efficient learning of perception tasks with minimal available data.

As for post-fusion, it occurs during the decision-making phase, where outputs from different modalities are combined to make final predictions. CLOCs [33] exemplify this approach; however, post-fusion methods tend to work with coarser granularity and often do not fully capitalize on the complementary strengths of point cloud and image data.

## 3. Method

**Figure 1:**
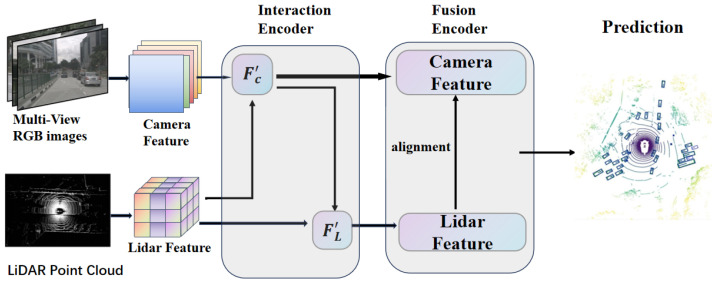
CrossInteraction overall framework: LiDAR feature and camera feature are taken as inputs. Interaction encoder: enhances this information through cross-modal interactions. Fusion encoder: integrates the enhanced features from both modalities before the prediction is completed.

In this study, we propose an improved modal interaction framework for multi-modal 3D object detection, termed CrossInteraction. An overview of our framework is illustrated in Figure 1. Initially, we extract representation information separately from the LiDAR and 2D image modalities. Within the interaction encoder, we conduct two sequential modal interactions. The first interaction enhances the image representation, which is then utilized as new input to improve the LiDAR representation in the second interaction. Subsequently, the fusion encoder integrates and aligns the enhanced representations of the two modalities. Specifically, $F_{c}^{\prime }$ denotes the images for post-interaction and $F_{L}^{\prime }$ represents the LiDAR for post-interaction.

### 3.1. Interaction Encoder

Before each modal interaction, the image modality representation $F_{c}$ and the LiDAR BEV modal representation $F_{L}$ are utilized as inputs. A dense map is implied to establish a pixel-level correspondence between these two representations. The depth map provides essential information about the distance of each pixel point in the scene, enabling to map both features into the same spatial domain. This mapping facilitates precise alignment and allows for the extraction of richer, more informative features. Consequently, we will create dense mappings between the image coordinate system and the BEV coordinate system, denoted as $M_{c\to L}$ and $M_{L\to c}$.

**Figure 2:**
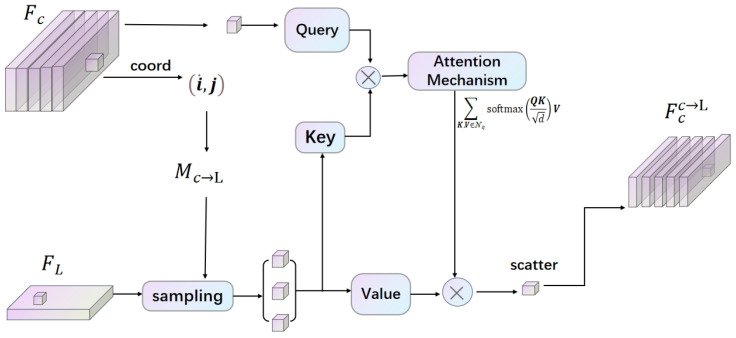
The first modal interaction: The LiDAR representation $F_{L}$ is employed to enhance the image representations $F_{c}$; this is processed by the attention machanism and we will then obtain the output $F_{c}^{c\to L}$.

**Figure 3:**
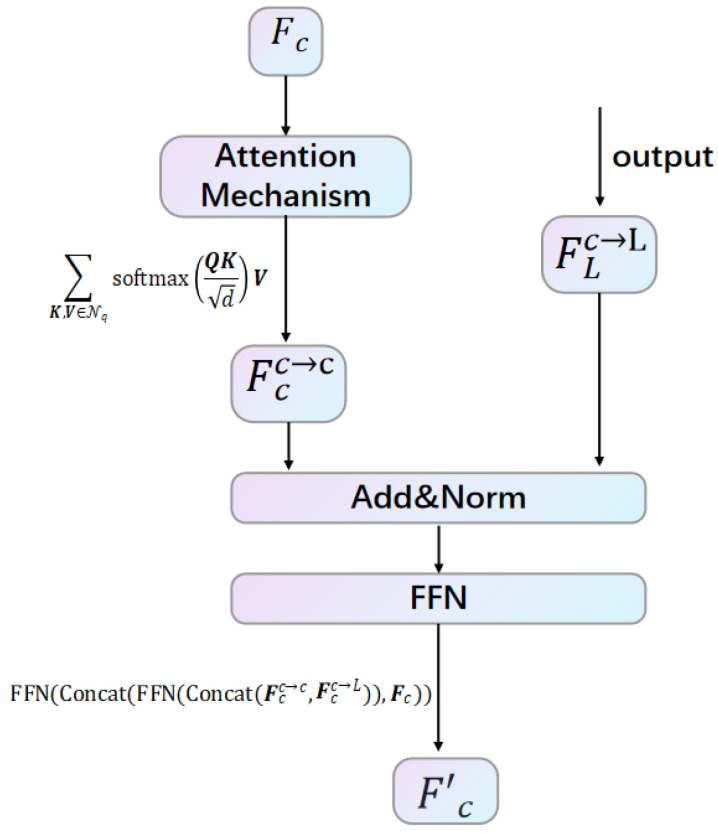
$F_{c}$ performs intra-modal representation learning a nd performs residual connection and normalization operations with the output $F_{c}^{c\to L}$; then, we obtain the final enhanced representation $F_{c}’$ through FFN.

In the first modal interaction, the framework is shown in Figure 2 and Figure 3. For $M_{c\to L}$ building and sampling, we begin by projecting the coordinates (x,y,z) of each point in the 3D point cloud onto the multi-camera setup to create a sparse depth map, referred to as $d_{sparse}$. Then, following the depth complementation method [34], we utilize this operation to obtain a dense depth map, denoted as $d_{dense}$. Using the dense depth map, we then project each pixel in the image space into the 3D point cloud space. For a given image pixel (i,j) with depth $d_{dense}^{\left[i,j\right]}$, the corresponding 3D coordinates (x,y,z) are obtained. We then use these 2D coordinates (x,y) to locate the corresponding BEV coordinates $\left(i_{L},j_{L}\right)$. This mapping is formalized as $T\left(i,j\right)=\left(i_{L},j_{L}\right)$, obtained through neighborhood sampling of size (2k+1)×(2k+1), which results in the correspondence $M_{c\to L}\left(i,j\right)$ = {T(i+Δi,j+Δj)|Δi,Δj∈[−k,+k]}.

Next, we employ an attention-based feature representation interaction. Each image point is treated as a query $Q=F_{c}^{\left[i_{c},j_{c}\right]}$, while the cross-modality neighbors $N_{q}=F_{L}^{\left[M_{c\to L}\left(i_{c},j_{c}\right)\right]}$ act as key *K* and value *V*; these are applied to achieve the cross-attention learning work:

(1) $$f_{\phi_{c\to L}}{\left(F_{c},F_{L}\right)}^{\left[i,j\right]}=\sum_{K,V\in N_{q}}\mathrm{softmax}\left(\frac{QK}{\sqrt{d}}\right)V,$$

where $F^{\left[i,j\right]}$ denotes the index of the element at position (i,j) in the 2D representation *F*. After $f_{\phi_{c\to L}}{\left(F_{c},F_{L}\right)}^{\left[i,j\right]}$ operation we get the initial LiDAR-enhanced image interaction feature $F_{c}^{c\to L}$.

At the same time, we apply the local attention, as described in Equation (1), utilizing a k×k grid neighborhood as the key *Q* and value *V* for both modalities. This further facilitates the learning of intra-modal representations. For image feature representation, we denote this process as $f_{\phi_{c\to c}}\left(F_{c}\right)$. Finally, through the representation integration framework, each layer outputs an enhanced image representation, which enriches the overall feature set for subsequent processing tasks.

(2) $$F_{c}^{\prime }=\mathrm{FFN}\left(\mathrm{Concat}\left(\mathrm{FFN}\left(\mathrm{Concat}\left(F_{c}^{c\to c},F_{c}^{c\to L}\right)\right),F_{c}\right)\right),$$

where FFN denotes feed-forward network, and Concat denotes concatenation operation.

**Figure 4:**
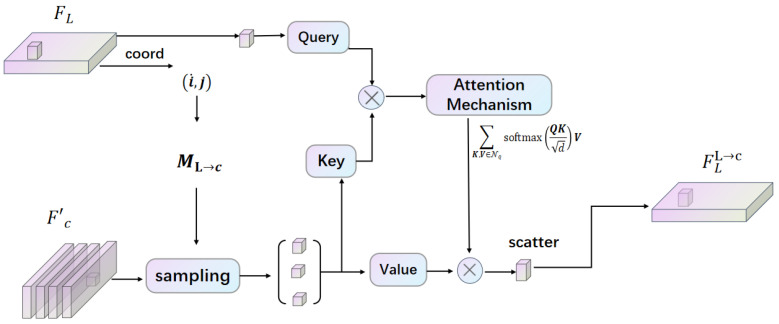
The second modal interaction: further enhancement of LiDAR representations $F_{L}$ using visual signals from enhanced image representations $F_{c}^{\prime }$; this is processed by the attention machanism and we will obtain the output $F_{L}^{L\to c}$.

**Figure 5:**
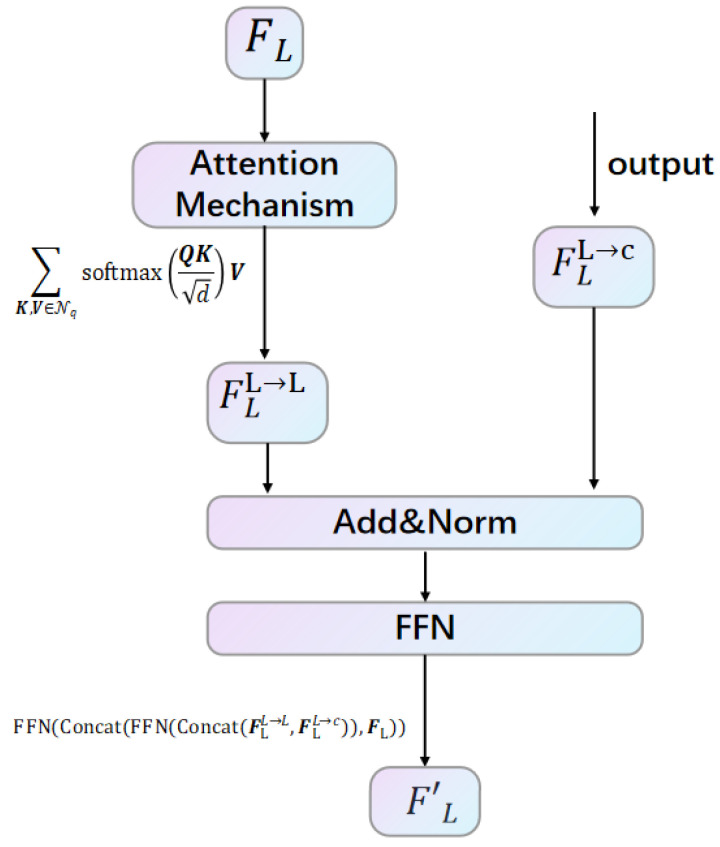
The second modal interaction: $F_{L}$ performs intra-modal representation learning and performs residual connection and normalization operations with the output $F_{L}^{L\to c}$; then, we obtain the final enhanced representation $F_{L}’$ through FFN.

In the second interaction, the framework is shown in Figure 4 and Figure 5. For $M_{L\to c}$ building and sampling, for LiDAR representations $F_{L}$, given the BEV coordinates $\left(i_{L},j_{L}\right)$, we first identify the LiDAR points {(x,y,z)} that correspond to the pillars at these coordinates. Subsequently, we project these 3D points into the camera image coordinate system to obtain the corresponding pixel coordinates {(i,j)}, which we denote as $M_{L\to c}\left(i_{L},j_{L}\right)=\left\{\left(i,j\right)\right\}$.

Similarly, during the attention-based feature interaction process, we take a LiDAR BEV point as the query, represented as $Q=F_{L}^{\left[i_{L},j_{L}\right]}$, and determine its cross-modality neighbors $N_{q}=F_{c}^{\prime [M_{c\to L}\left(i_{L},j_{L}\right)]}$. We apply the same mechanism outlined in Equation (1) to facilitate this second interaction, encapsulated as $f_{\phi_{L\to c}}{\left(F_{L},F_{c}^{\prime }\right)}^{\left[i,j\right]}$. Furthermore, while learning the intra-modal representations, we utilize $f_{\phi_{L\to L}}\left(F_{L}\right)$ for the LiDAR feature representation. The final output of the representation integration following the second interaction is obtained as $F_{L}^{\prime }$, which enhances the overall feature set for subsequent tasks. Here, define $F_{L}^{\prime }$ as

(3) $$F_{L}^{\prime }=\mathrm{FFN}\left(\mathrm{Concat}\left(\mathrm{FFN}\left(\mathrm{Concat}\left(F_{L}^{L\to L},F_{L}^{L\to c}\right)\right),F_{L}\right)\right),$$

### 3.2. Fusion Encoder

#### 3.2.1. Feature Alignment

Graph Convolutional Network Formula:

(4) $$H^{\left(l+1\right)}=\sigma \left({\tilde{D}}^{-\frac{1}{2}}\tilde{A}{\tilde{D}}^{-\frac{1}{2}}H^{\left(l\right)}W^{\left(l\right)}\right),$$

where σ denotes the nonlinear activation function, $H^{\left(l\right)}$ is the node feature matrix of layer l, and $H^{\left(l+1\right)}$ indicates the node feature matrix of layer l+1. The matrix $\tilde{A}$ denotes the adjacency matrix with self-connections, $\tilde{D}$ denotes the degree matrix associated with the adjacency matrix that includes these self-connections, and $W^{\left(l\right)}$ denotes the learning weight matrix for layer l.

**Figure 6:**
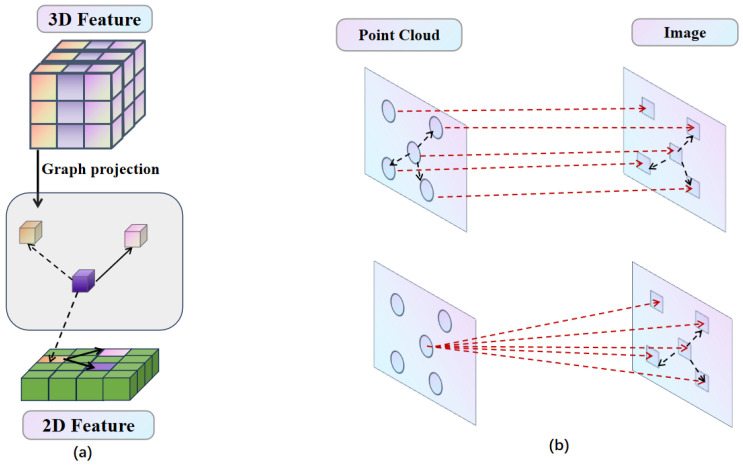
Alignment Framework: (**a**) shows our alignment framework uses graph-based feature alignment to match more reasonable alignments between modalities. (**b**) shows two methods we used to project the point cloud features onto the image with one-to-one and one-to-many within alignment process, which can achieve better alignment, in order to achieve better results in the subsequent perception training tasks.

Projection is limited by sensor errors, while attention mechanisms require massive computation. To address these issues, we use the graph convolutional network method, as shown in Figure 6, and we project point cloud features onto image features to identify the *K* nearest neighbors of these image features. We then perform one-to-many fusion by integrating each individual point cloud feature with its *K* neighboring image features, thereby enhancing the alignment between the two modalities.

#### 3.2.2. Feature Fusion

**Figure 7:**
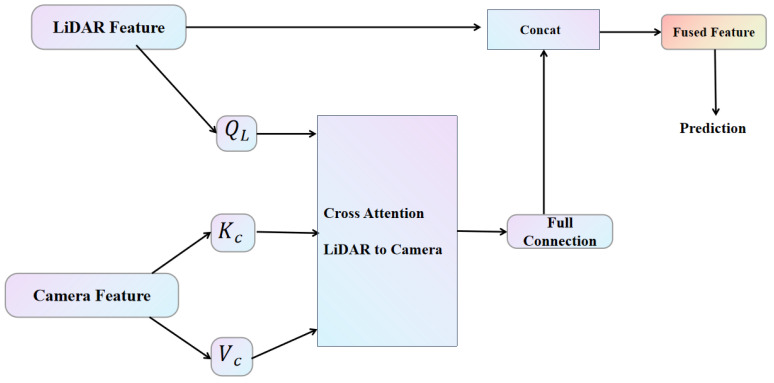
Fusion framework: LiDAR BEV features are utilized as input to query module $Q_{L}$; the camera image features are transformed into both the key module $K_{c}$ and the value module $V_{c}.$

The fusion framework is shown in Figure 7. To effectively integrate features from LiDAR and camera images, we introduce a cross-attention mechanism that enhances the understanding of the interrelationships between these two modalities. In the deep feature fusion step, we first transform each LiDAR feature into a voxel representation containing multiple points, while associating the corresponding camera pixels with a polygonal region. This input data structure comprises a voxel unit and its associated *N* camera features. We design three fully-connected layers to convert the voxel features into a query module ($Q_{L}$) and the camera features into a key module ($K_{c}$) and a value module ($V_{c}$), respectively. For each query (i.e., voxel unit), an attentional affinity matrix is generated by performing an inner product operation between the query module and the key module, which reflects the correlation between the 1N voxel units and their associated *N* camera features. This attentional affinity matrix is subsequently employed to weight and aggregate the $V_{c}$ values, which encapsulate the camera information, by applying the softmax operator for normalization. The fused camera information is then processed through a fully connected layer and combined with the original LiDAR features. The resulting fused features are suitable for input into any standard 3D detection framework, effectively leveraging the complementary information from both modalities to enhance detection performance.

## 4. Experiments

### 4.1. Introduction of Dataset

The nuScenes dataset covers about 1.4 million camera images, 400,000 Ladar scans, and 1.4 million millimetre-wave radar scans. nuScenes selects 40,000 keyframes and annotates about 1.4 million 3D target bounding boxes, which is seven times greater than that of the KITTI dataset. The scenes are categorized into four distinct types based on environmental conditions: daytime, nighttime, sunny days, and rainy days. nuScenes support multiple tasks including detection, tracking, and BEV map segmentation. It contains driving scenarios collected in Boston and Singapore. These two cities are known for their dense traffic and challenging driving environments. Such scenes include high traffic density (e.g., intersections, construction sites), rare classes (e.g., ambulances, animals), potentially dangerous traffic situations (e.g., jaywalkers, incorrect behavior), maneuvers (e.g., lane change, turning, stopping), and difficult situations.

For evaluation purposes, nuScenes utilizes traditional metrics such as mean average precision (mAP) while also introducing the exclusive nuScenes detection score (NDS). The NDS is calculated as a weighted average of five true positive (TP) metrics: the mean translational error (mATE), which measures the 2D center distance between the predicted and true values using the Euclidean distance; the mean scale error (mASE), which quantifies the difference between threshold 1 and the 3D intersection and merger ratio; the mean angular error (mAOE), representing the smallest yaw angle difference between the predicted and true values; the mean velocity error (mAVE), which is the L2 norm of the 2D velocity difference; and the mean attribute error (mAAE), which indicates the difference between 1 and the category classification accuracy. By integrating these metrics, the NDS provides a comprehensive evaluation of the detection performance. The formula is shown below:

(5) $$\mathrm{NDS}=\frac{1}{10}\left[5mAP+\sum_{mTP\in \mathrm{TP}}\left(1-\min\left(1,mTP\right)\right)\right],$$

MACs serve as a measure of computational complexity, indicating the total number of multiply–add operations required by the model to process data. A higher MAC count signifies greater model complexity and increased computational demand. Latency, on the other hand, refers to the time elapsed from the input of data to the generation of output results. Lower latency indicates a faster model response, which is particularly critical in real-time applications such as autonomous driving, where it directly impacts system responsiveness and safety.

### 4.2. Implementation Details

In this study, we evaluate the performance of BEVFusion [5] in the integration of camera and LiDAR data for 3D object detection and BEV map segmentation, addressing both geometric and semantic challenges. Our architecture is designed to be modular, which facilitates the incorporation of additional sensor types, such as radars and event-based cameras, and allows adaptation to various 3D perception tasks, including 3D object tracking and motion prediction.

The model employs the Swin Transformer as the image processing backbone and VoxelNet for processing LiDAR data. To integrate features at multiple scales from the camera, we utilize a Feature Pyramid Network (FPN), producing a feature map that is **1/8** the size of the original input. The camera images are downsampled to a resolution of **256 × 704** pixels, while the LiDAR point cloud is voxelized at resolutions of **0.075** m for detection tasks and **0.1** m for segmentation tasks. Given that detection and segmentation require different spatial coverage and feature map sizes, we implement grid sampling with bilinear interpolation before each task-specific module to facilitate the transition between various BEV feature maps.

To enhance feature alignment and ensure consistency across sensor data, we build upon cross-attention mechanisms. We conceptualize the LiDAR point cloud and camera features as nodes within a graph, establishing graph edges based on spatial correlations and feature affinities. This approach allows for a more precise and coherent integration of multi-sensor data, optimizing the performance of 3D perception tasks.

Training procedure: In contrast to existing methods that typically freeze the camera encoder [2,23,25], we adopt an end-to-end training strategy for the entire model. To mitigate overfitting, we apply data augmentations to both the image and LiDAR modalities. The model optimization is performed using the AdamW optimizer with a weight decay of **$10^{-2}$**.

Setup: We use the mAP across 10 foreground classes and the NDS as our detection metrics. Additionally, we measure the single-inference #MACs and latency on an RTX3090 GPU for all open-source methods. We employ a single model without any test-time augmentation for both val and test results.

### 4.3. Results and Analysis

We achieve state-of-the-art results on the nuScenes detection benchmark, as shown in Table 1. Compared with BEVfusion [5], we achieve a 1.9% increase in mAP and a 0.9% increase in NDS, while reducing the number of MACs to **249 G** and measuring latency at **110.9 ms**. Additionally, we compare favorably against representative point-level fusion methods, such as PointPainting [2] and MVP [3], with about **1.7** × speedup, **1.5** × reduction in MACs, and **4.5%** increase in mAP on the val set. In comparison with other state-of-the-art methods [35,36,37], our method can still maintain its advantages. The efficiency gains observed in our CrossInteraction method can be attributed to the dual interaction of modality features, which maximizes the utilization of camera features. This optimization allows us to maintain competitive performance with significantly lower MACs.

Table 2 shows the results of BEV map segmentation, where our camera-only model demonstrates superior performance, outperforming LiDAR-only baselines by **8–13%**.

We further conduct a systematic analysis of CrossInteraction’s performance under varying weather and lighting conditions, as illustrated in Table 3. Detecting objects in rainy conditions poses significant challenges for LiDAR-only models due to pronounced sensor noise. However, due to the inherent robustness of camera sensors across different weather situations, our model enhances CenterPoint by **6.3** mAP and exceeds BEVfusion [5] by **1+** mAP, effectively bridging the performance gap between sunny and rainy scenarios. Lighting conditions also present challenges for all models. In daytime scenarios, we achieve a mAP exceeding **70**, surpassing BEVfusion by **2.4** mAP. The performance of camera-only models significantly deteriorates under nighttime conditions. Despite this, our multi-sensor fusion approach still exceeds BEVfusion by **1.4** mAP and achieves an improvement of **17.1** mIoU compared to CentrePoint. This gain is not only substantial but also emphasizes the importance of geometric cues in scenarios where camera sensors may underperform.

The ablation studies presented in Table 4, Table 5, Table 6, Table 7 and Table 8 serve to validate our design decisions. Specifically, in Table 4, we evaluate the effect of using different numbers of representations/modalities in decoding. We compare our multi-modal interaction framework using both representations in an alternating manner with a variant using LiDAR representation in all decoder layers. The results demonstrate that using both representations is beneficial, validating our design rationale. Table 5 highlights the effectiveness of our detection algorithm in scaling to voxel-based representations, achieving the best balance between accuracy and efficiency with a voxel size of 0.075. Table 6 shows that our model is universal and applicable to different backbone networks. Notably, the common practice to freeze the image backbone in existing multi-sensor 3D object detection research does not exploit the full potential of the camera feature extractor. Table 7 indicates that increasing the number of encoder layers up to three layers can consistently improve the performance whilst introducing negligible negative impact, validating the necessity of multi-layer encoders for multi-modal feature fusion. In Table 8, for a fair comparison, both methods employ the same number of encoder layers as well as the same decoder. From a one-way interaction perspective, the mAP of LiDAR-enhanced camera features is lower than that of camera-enhanced LiDAR features. This discrepancy may stem from the inherent stability of LiDAR’s point cloud coordinate system, which enhances visual semantics with geometric precision. Overall, our representation interaction method is more effective than the one-way interaction approach.

In order to verify the generalization and stability of the model, we set up cross-dataset experiments, where we add the Waymo dataset to the original nuScenes dataset. The Waymo dataset is much larger than the original dataset and contains a large amount of high-quality data, especially high-frequency annotations. We let the two datasets be used as the training set and test set for experiments, respectively, and compare them with BEVFusion [5] to obtain the experimental results in Table 9. From the results, we can see from mAP that our model outperforms BEVFusion [5] in terms of generalization and stability. Unlike BEVFusion’s tightly coupled fusion approach, CrossInteraction ensures the independence of LiDAR BEV features and camera panoramic features. This design preserves the advantages of each modality even when the test set distribution shifts, avoiding the information loss caused by BEVFusion’s forced unified representation. For example, when the sparsity of the LiDAR point cloud in the test set (NuScenes) is significantly higher than that in the training set (Waymo), the interaction weight of the camera features is automatically increased to compensate for the lack of geometric information, thereby reducing mAP fluctuation. Furthermore, through the $M_{L\to c}$ and $M_{c\to L}$ mapping matrices mentioned in Section 3.1 of the article, pixel-level alignment in BEV space is established, enforcing the geometric prior trained on Waymo to adapt to the differences in sensor configuration in NuScenes (such as changes in camera perspective), reducing the drop in mAP caused by shifts.

### 4.4. Visualizations

**Figure 8:**
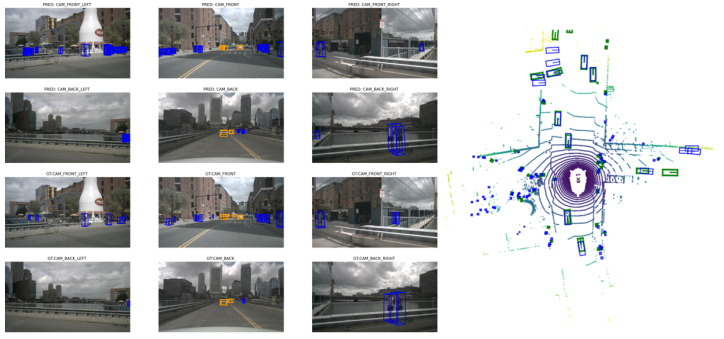
Qualitative results on nuScenes val set. In the camera image (**left**), "pred" represents the algorithm’s prediction, "GT" represents the ground truth, blue boxes represent pedestrians, and yellow boxes represent cars. In LiDAR BEV (**right**), green boxes are the ground-truth and blue boxes are the predictions.

Figure 8 shows the qualitative results of our multimodal representation interaction algorithm for 3D object detection and perception on the nuScenes validation set. Left is the camera image and right is LiDAR BEV. As can be seen, the camera can capture visual features such as color and texture, covering a wider field of view and providing rich semantic information, which facilitates the recognition of objects such as pedestrians and vehicles. The LiDAR BEV directly acquires 3D coordinates and depth information through laser scanning, unaffected by lighting conditions and clearly reflecting geometric features such as object shape and size. This provides important insights for future collaboration on our algorithm optimization and hardware adaptation.

**Figure 9:**
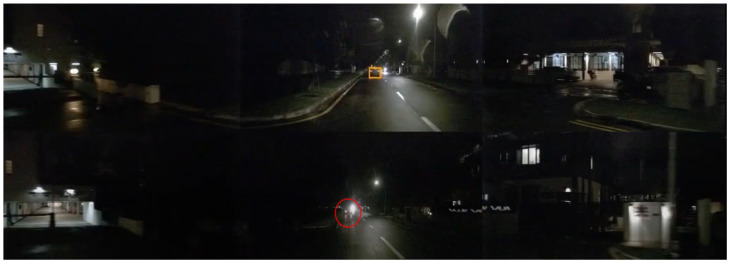
Visualization of night scene recognition failure case.

As shown in Figure 9, it can be seen that in low-light conditions such as at night, motion blur and increased inter-frame noise can affect the effectiveness of BEV temporal fusion, weakening the ability to ”visually complete” objects in distant areas.

## 5. Discussion

The fusion of multi-modal information can overcome the shortcomings of single-modality 3D object detection, thereby improving performance and accuracy. In real-world automotive systems, more robust 3D perception methods like our CrossInteraction model are expected to be further optimized and deployed within the perception modules of autonomous driving systems. This, combined with higher prediction accuracy and lower latency, could reduce the potential for accidents, thereby improving the safety and reliability of autonomous driving. However, multi-modal algorithms typically demand more computational requirements and higher operational costs to run. This raises the need for future improvements in system efficiency.

## 6. Conclusions

In this paper, we propose an enhanced approach to 3D object detection called CrossInteraction. This method focuses on preserving and enhancing the representational information of two modalities: images and LiDAR. Specifically, it strengthens the output of the image representation to serve as the mapping input for the LiDAR data, facilitating deeper interaction through representational learning. By leveraging representational learning, CrossInteraction effectively tackles the challenge of multi-modal data fusion, where the strengths of individual modalities are often underutilized—particularly the image representation, due to the auxiliary source role processing. To overcome the problem of misaligned modal feature information, we utilize a graph convolutional network to avoid sensor accuracy errors. Additionally, pre-training with Transformers is employed to reduce overall training time, thereby improving the efficiency of the approach. Experiments demonstrate that our model achieves superior performance on both nuScenes 3D detection and BEV map segmentation tasks.

Limitations: Our multi-modal fusion components are agnostic to modality-specific representations. The explicit 2D-3D mapping relies heavily on precise sensor calibration. Alignment via graph convolutional networks (GCNs) alone may lose significant modality-specific information due to compression artifacts. Future work could integrate attention mechanisms to learn adaptive cross-modal alignment.

As shown in Figure 9, our method fails to mitigate camera failures under strong nighttime illumination (e.g., headlight glare). Thus, in the future, we also need to investigate if camera distortion propagates to LiDAR features via cross-modal interaction and to validate performance in extreme weather (e.g., snow, sandstorm).

Finally, processing dual-sensor data streams and maintaining multi-modal features incur significant computational overhead. Future work will develop an adaptive framework to dynamically select interaction operators, optimizing the trade-off between performance, efficiency, and robustness.

## Figures and Tables

**Table 1:** The results of 3D Object Detection. ^†^: w/ test-time augmentation (TTA); ^‡^: w/ model ensemble and TTA; ‘L’ and ‘C’ represent LiDAR and camera, respectively.

	Modality	mAP (*test*)	NDS (*test*)	mAP (*val*)	NDS (*val*)	MACs (G)	Latency (ms)
BEVDet [10]	C	42.2 ^†^	48.2 ^†^	–	–	–	–
M^2^BEV [38]	C	42.9	47.4	41.7	47.0	–	–
BEVFormer [17]	C	44.5	53.5	41.6	51.7	–	–
BEVDet4D [11]	C	45.1 ^†^	56.9 ^†^	–	–	–	–
PointPillars [21]	L	–	–	52.3	61.3	65.5	34.4
SECOND [19]	L	52.8	63.3	52.6	63.0	85.0	69.8
CenterPoint [22]	L	60.3	67.3	59.6	66.8	153.5	80.7
PointPainting [2]	C+L	–	–	65.8	69.6	370.0	185.8
PointAugmenting [23]	C+L	66.8 ^†^	71.0 ^†^	–	–	408.5	234.4
MVP [3]	C+L	66.4	70.5	66.1	70.0	371.7	187.1
FusionPainting [39]	C+L	68.1	71.6	66.5	70.7	–	–
AutoAlign [40]	C+L	–	–	66.6	71.1	–	–
FUTR3D [41]	C+L	–	–	64.5	68.3	1069.0	321.4
TransFusion [25]	C+L	68.9	71.6	67.5	71.3	485.8	156.6
BEVfusion [5]	C+L	**70.2**	**72.9**	**68.5**	**71.4**	**253.2**	**119.2**
VoxelNeXt [35]	C+L	**70.3**	**72.2**	**68.6**	**71.7**	**777.3**	**234.2**
FusionPlanner [36]	C+L	**69.5**	**71.9**	**67.2**	**70.3**	**459.4**	**210.4**
LaneGAP [37]	C+L	**70.2**	**71.4**	**68.2**	**70.9**	**542.2**	**220.1**
Ours	C+L	**72.1**	**73.8**	**70.3**	**72.2**	**249.0**	**110.9**
VoxelNeXt [35]	C+L	**74.4** ^‡^	**75.4** ^‡^	–	–	–	–
FusionPlanner [36]	C+L	**74.6** ^‡^	**75.9** ^‡^	–	–	–	–
LaneGAP [37]	C+L	**74.2** ^‡^	**76.0** ^‡^	–	–	–	–
BEVfusion [5]	C+L	**75.0** ^‡^	**76.1** ^‡^	**73.7** ^‡^	**74.9** ^‡^	–	–
Ours	C+L	**76.3** ^‡^	**77.4** ^‡^	**74.2** ^‡^	**76.3** ^‡^	–	–

**Table 2:** The results of BEV map segmentation. ‘L’ and ‘C’ represent LiDAR and camera, respectively.

	Modality	Drivable	Ped. Cross.	Walkway	Stop Line	Carpark	Divider	Mean
OFT [9]	C	74.0	35.3	45.9	27.5	35.9	33.9	42.1
LSS [7]	C	75.4	38.8	46.3	30.3	39.1	36.5	44.4
CVT [42]	C	74.3	36.8	39.9	25.8	35.0	29.4	40.2
M^2^BEV [38]	C	77.2	–	–	–	–	40.5	–
PointPillars [21]	L	72.0	43.1	53.1	29.7	27.7	37.5	43.8
CenterPoint [22]	L	75.6	48.4	57.5	36.5	31.7	41.9	48.6
PointPainting [2]	C+L	75.9	48.5	57.1	36.9	34.5	41.9	49.1
BEVFusion [5]	C+L	**85.5**	**60.5**	**67.6**	**52.0**	**57.0**	**53.7**	**62.7**
VoxelNeXt [35]	C+L	**85.4**	**57.2**	**63.4**	**53.7**	**55.9**	**54.7**	**61.7**
LaneGAP [37]	C+L	**82.2**	**50.4**	**59.0**	**52.1**	**54.6**	**52.0**	**58.4**
Ours	C+L	**88.7**	**62.4**	**68.2**	**53.9**	**59.8**	**54.9**	**64.7**

**Table 3:** The results of robust experiment. ‘L’ and ‘C’ represent LiDAR and camera, respectively.

		Sunny			Rainy			Day			Night	
	Modality	mAP	mIoU		mAP	mIoU		mAP	mIoU		mAP	mIoU
CenterPoint [22]	L	62.9	50.7		59.2	42.3		62.8	48.9		35.4	37.0
BEVDet [10]	C	32.9	59.0		33.7	50.5		33.7	57.4		13.5	30.8
MVP [3]	C+L	65.9 (+3.0)	51.0 (−8.0)		66.3 (+7.1)	42.9 (−7.6)		66.3 (+3.5)	49.2 (−8.2)		38.4 (+3.0)	37.5 (+6.7)
BEVFusion [5]	C+L	68.2 (+5.3)	65.6 (+6.6)		69.9 (+10.7)	55.9 (+5.4)		68.5(+5.7)	63.1 (+5.7)		42.8(+7.4)	43.6 (+12.8)
Ours	C+L	69.2 (+6.3)	68.9 (+9.9)		72.0 (+12.8)	58.3 (+7.6)		70.9(+8.1)	65.2 (+7.8)		44.2(+8.8)	47.9 (+17.1)

**Table 4:** Ablation experiments and default settings (modality). ‘L’ and ‘C’ represent LiDAR and camera, respectively.

	mAP	NDS	mIoU
L	58.4	66.7	57.1
C+L	**72.1**	**72.2**	**64.7**

**Table 5:** Ablation experiments and default settings (voxel size).

(Meters)	mAP	NDS	mIoU
0.075	**72.1**	**72.2**	**64.7**
0.1	67.5	71.5	64.3
0.125	67.2	70.5	64.4

**Table 6:** Ablation experiments and default settings (feature interaction backbone).

	mAP	NDS	mIoU
ResNet50	67.1	68.7	60.2
SwinT (freeze)	66.3	71.2	54.0
SwinT	**72.1**	**72.2**	**64.7**

**Table 7:** Ablation experiments and default settings (interaction encoder layers).

	mAP	NDS
1	66.3	70.6
2	67.5	71.4
3	**69.8**	**72.7**

**Table 8:** Ablation experiments and default settings (each modal interaction).

	mAP	NDS
$F_{L\to c}$	68.3	70.5
$F_{c\to L}$	69.5	71.9
$F_{L\to c}$ + $F_{c\to L}$	**72.1**	**72.2**

**Table 9:** Cross-dataset test experiments.

	mAP (nuScenes→Waymo)	mAP (Waymo→nuScenes)
BEVFusion [5]	67.3	47.5
Ours	**72.1**	**56.9**

## Data Availability

The original contributions presented in this study are included in the article. NuScenes and Waymo are publicly available datasets and were used in this study. available online: https://www.nuscenes.org/nuscenes (access on 10 March 2024); available online: https://waymo.com/open/download/ (access on 10 March 2024).
